# COVID-19 and oral lesions, short communication and review

**DOI:** 10.4317/jced.57981

**Published:** 2021-03-01

**Authors:** Sonia Egido-Moreno, Joan Valls-Roca-Umbert, Enric Jané-Salas, José López-López, Albert Estrugo-Devesa

**Affiliations:** 1 DDS. Professor of Oral Pathology. Department of Odontostomatology. Faculty of Medicine and Health Sciences (Dentistry), University of Barcelona. University Campus of Bellvitge, Barcelona, Spain; 2 DDS, Professor of Master’s degree, School of Dentistry, University of Barcelona. University Campus of Bellvitge, Barcelona, Spain; 3 PhD, DDS, MD. Professor of Oral Pathology. Department of Odontostomatology. Faculty of Medicine and Health Sciences (Dentistry), University of Barcelona. University Campus of Bellvitge, Barcelona, Spain / Oral Health and Masticatory System Group (Bellvitge Biomedical Research Institute) IDIBELL, University of Barcelona, L’Hospitalet de Llobregat, Barcelona, Spain; 4 DDS, MD, PhD, Department of Odontoestomatology. Faculty of Medicine and Health Sciences (School of Dentistry), University of Barcelona. University Campus of Bellvitge, Barcelona, Spain. / Dental Hospital University of Barcelona, (Barcelona University) / Oral Health and Masticatory System Group (Bellvitge Biomedical Research Institute) IDIBELL, Barcelona, Spain

## Abstract

**Background:**

The COVID-19 disease first appeared in December 2019 in Wuhan, China. The World Health Organization (WHO) declared the pandemic in March 2020, with 40 million cases and a million deaths in October 2020. COVID-19 also includes manifestations on the skin and mucous mucosal membrane. Objective: To evaluate the prevalence of the oral lesions associated to COVID-19 disease; and evaluate their clinical presentation and the hypothesized etiology.

**Material and Methods:**

An electronic literature search was performed in PubMed, Scopus and Índice Médico Español databases. The following combination of keywords and Boolean operators were used: “COVID-19 AND oral manifestations”; “COVID-19 AND oral lesions”; “COVID-19 AND mucosal lesions” ; “COVID-19 AND mucosal manifestations”; “SARS-COV-2 AND oral manifestations”; “SARS-COV-2 AND oral lesions”; “SARS-COV-2 AND mucosal lesions”; “SARS-COV-2 AND mucosal manifestations”. Furthermore, the bibliography was reviewed to manually include additional articles. The risk of bias in individual studies was assessed by two blinded reviewers using the Joanna Briggs Institute (JBI) and the evidence levels of the articles found will be cataloged according to the level of evidence and grade of recommendation of Oxford Centre for Evidence-Based Medicine (CEBM).

**Results:**

249 articles were found in the Medline / Pubmed database. There are no additional articles in the Scopus and Índice Médico Español databases. We selected 14 articles plus 5 more articles due to manual searching. Patients presented a wide variety of oral manifestations. The most prevalent were lesions with a solution of continuity (n = 48, 73.85%) and the most frequent area was the tongue (n = 41, 52.56%). The preferred treatment for the lesions is a localized one by using rinses.

**Conclusions:**

To conclude, after the bibliographic review was performed, we can expect that the COVID-19 disease can cause cutaneous and mucosal lesions as secondary manifestations. Despite more studies being needed to confirm this.

** Key words:**COVID-19, SARS-COV-2, oral lesions, oral manifestations.

## Introduction

The COVID-19 disease first appeared in December 2019 in Wuhan, China (1). The World Health Organization (WHO) declared the pandemic in March 2020, with confirmed cases in October 2020 that exceeded 40 million affected and more than 1 million deaths worldwide (2). The most common symptoms associated with this viral infection are: fever, cough, headache, diarrhea, fatigue, and myalgia (1,3).

COVID-19 also includes manifestations on the skin and mucous mucosal membranes (4). To date, few descriptions of the cutaneous manifestations of COVID-19 and few clinical images have been published due to safety reasons (4,5). Erythematous eruptions, generalized urticaria, or chickenpox-like vesicles are especially present (5). On the other hand, in April 2020 Martín Carreras-Presas *et al.* (6) published the first work on oral manifestations associated with the disease; since then different publications have referred to the subject. Given that the oral health of COVID-19 patients can be affected by infection, there are still doubts as to whether these manifestations could be a typical pattern resulting from direct viral infection, caused by systemic deterioration, considering the possibility of opportunistic infections, or adverse reactions to treatments (7).

The aim of this work is to evaluate the prevalence of oral lesions associated with COVID-19 disease; and assess the clinical presentation and its etiological hypothesis.

## Material and Methods

An electronic literature search was performed in PubMed, Scopus and Índice Médico Español databases.

Articles were selected by two blinded reviewers (SEM and JVR). Firstly, they reviewed titles and abstracts (phase-1). If papers were considered eligible for inclusion, a full-text reading was blindly performed by the same reviewers (phase-2). In case of disagreements, a third reviewer (AED) was involved to make the final decision and agreed upon with JLL. The reporting of these systematic review followed the Preferred Reporting Items for Systematic Review and Meta-Analyses (PRISMA) (8) guidelines for adequate conductance of systematic reviews.

The following combination of keywords and Boolean operators were used: “COVID-19 AND oral manifestations”; “COVID-19 AND oral lesions”; “COVID-19 AND mucosal lesions” ; “COVID-19 AND mucosal manifestations”; “SARS-COV-2 AND oral manifestations”; “SARS-COV-2 AND oral lesions”; “SARS-COV-2 AND mucosal lesions”; “SARS-COV-2 AND mucosal manifestations”. Furthermore, the bibliography was reviewed to manually include additional articles.

The following inclusion criteria were applied: Studies that were only related with SARS-COV2; No language or publication date restrictions; Humans; Case series, case studies or letters to the editor.

The risk of bias in individual studies was assessed by two blinded reviewers using the Joana Briggs Institute (JBI) (9) critical appraisal checklist for case reports. The answers could be “yes”, “unclear”, “no”, or “not applicable”. Decisions about scoring were agreed upon by all reviewers before the critical appraisal commenced, and studies were characterized according to the following: (a) low risk of bias, if studies reached more than 70% scores of “yes”; (b) moderate risk of bias, if “yes” scores were between 50% and 69%; and (c) high risk of bias, if “yes” scores were below 49% (9). The evidence levels of the articles found will be cataloged according to the level of evidence and grade of recommendation of Oxford Centre for Evidence-Based Medicine (CEBM) (10) (Table 1).

**Table 1:**
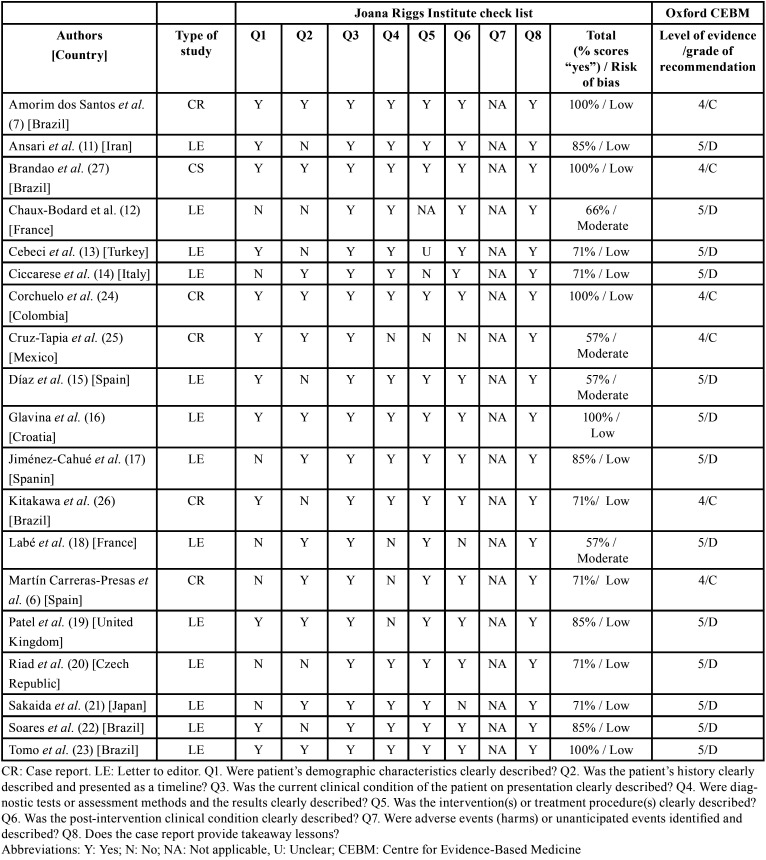
Study characteristics and risk of bias assessed by the Joanna Briggs Institute critical appraisal tool for case reports and, level of evidence and grade of recommendation of Oxford Centre for Evidence-Based Medicine (10).

## Results

**Figure 1:**
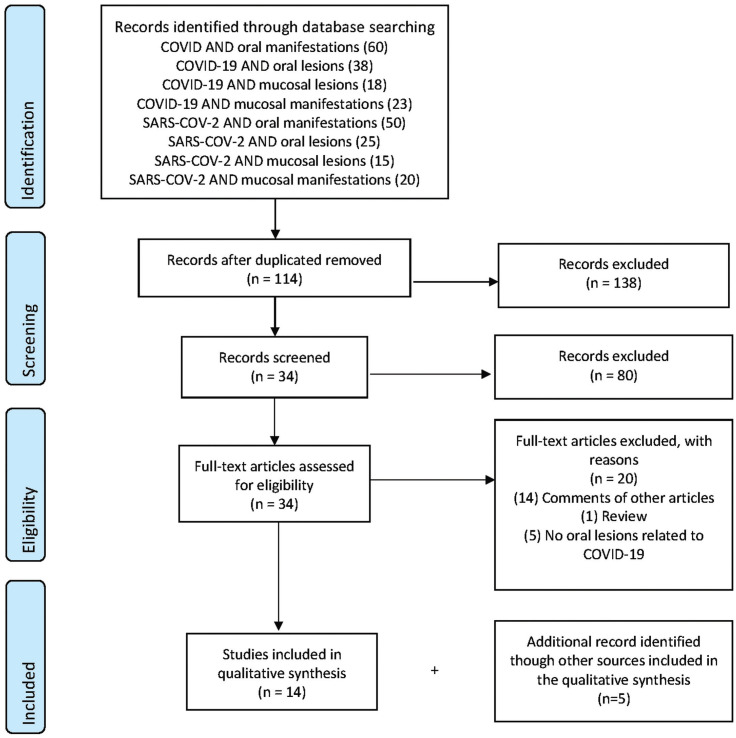
Flow chart of search strategy in Pubmed.

Using our search strategy, 249 articles were found in the Medline / Pubmed database. There are no additional articles in the Scopus and Spanish Medical Index databases. Of the 249 articles initially found, 138 were excluded because they were duplicates. After reading the titles and abstracts, 80 were excluded because they did not meet the inclusion and exclusion criteria. Of these, the remaining 34 studies were read in full text and 17 articles were deleted because they were only comments of other articles (14), reviews (1) or the articles did not mention a relationship between oral lesions and COVID-19 disease (5). Finally, we included 14 articles for this synthesis and added 5 more studies by manual search (Fig. 1).

With regard to the risk of bias in the individual studies based on CEBM, most were letters to the editor (11-23) and case reports (6,7,24-27), therefore the level of evidence and the grade of recommendation is low. Despite the low evidence we included these articles due to the lack of available data until this time. The risk-of-bias in individual studies is also assessed with JBI critical appraisal checklist for case reports. Most were judged as low risk (6,7,11,13-17,19-24,26,27), and three as a moderate risk (12,18,25) (Table 1).

The characteristics of patients and lesions are summarized in Table 2,  Table 2 cont. The total population included was of 60 patients, comprised of 24 men (40%) and 36 women (60%). The medium age of the patients was of 41.26±19.05 years. PCR test was positive to SARS-COV-2 (confirming the diagnosis of COVID-19) in 55 patients (91.67%) and for 5 patients the diagnosis was probably because of the symptoms but a test was not performed to confirm this suspicion.

**Table 2:**
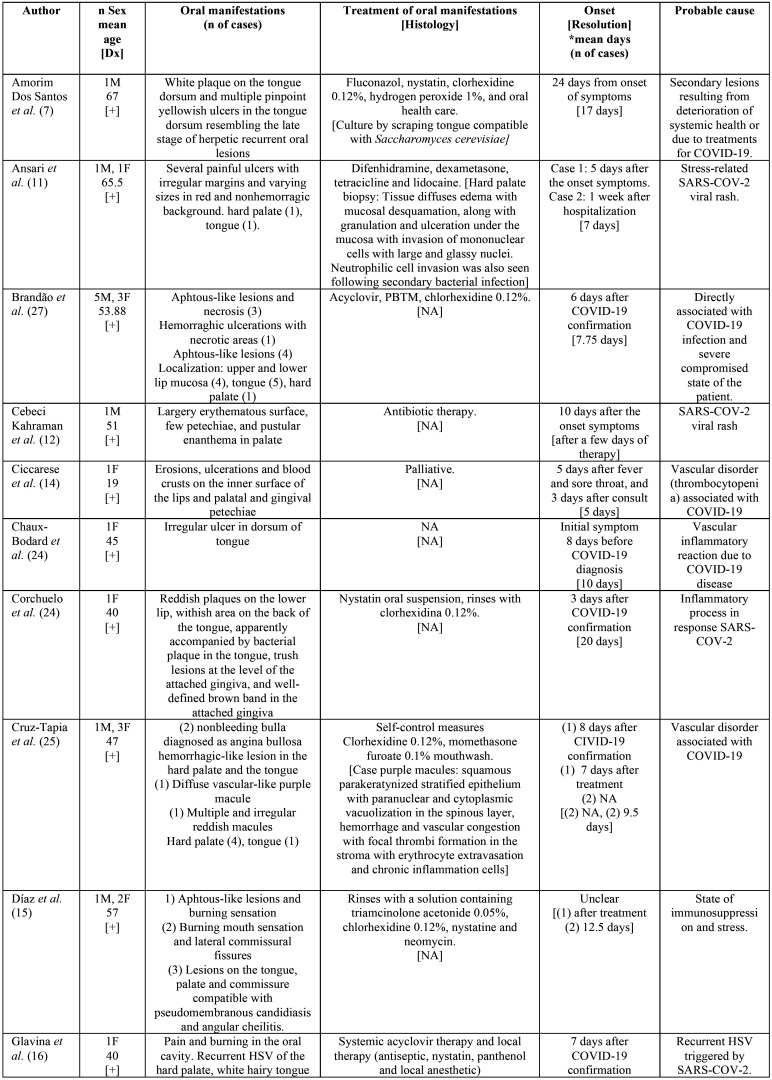
Characteristics of the patients and lesions.

**Table 2 cont.**
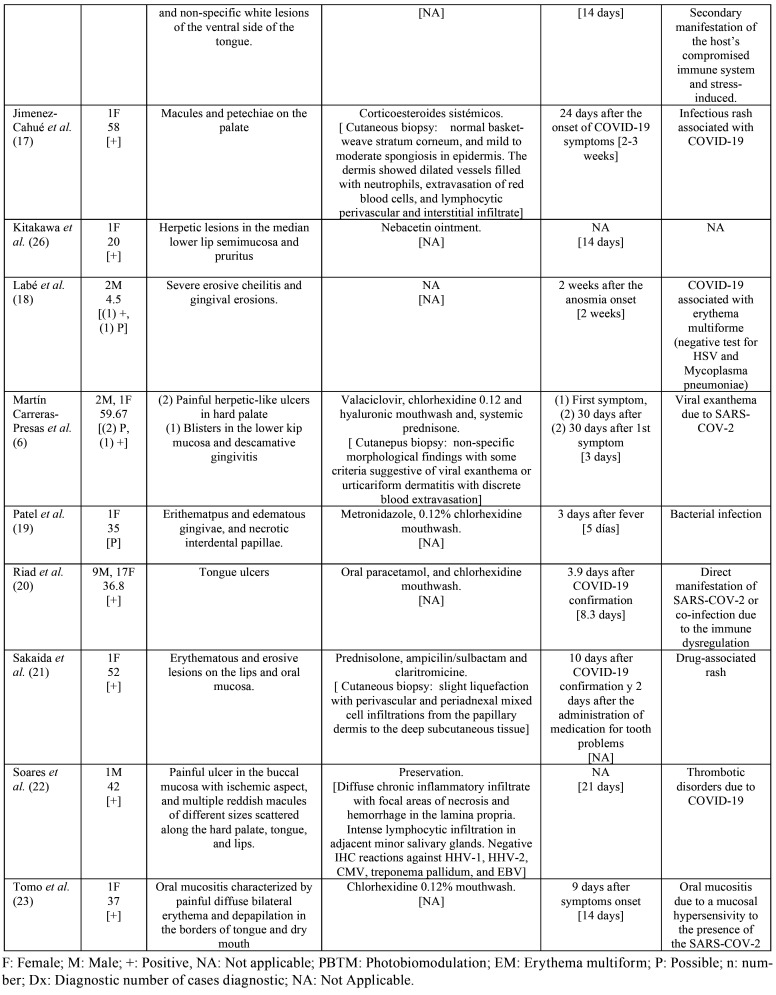
Characteristics of the patients and lesions.

Patients presented a wide variety of oral manifestations. The most prevalent were lesions with a solution of continuity (n = 48, 73.85%) such as ulcers, aphtous-like lesions or erosions (n = 36, 55.38%; n = 8, 12.31%; n = 4, 6.15% respectively), followed by macules (n = 4, 6.15%) and petechiae (n = 3, 4.61%), plaques (n = 3; 4.61%), bullae (n = 2, 3.08%); gingival abnormalities, such as desquamative and necrotizing gingivitis (n = 1, 1.54% both) and finally blisters and pustules (n = 1, 1.54% both).

The most frequent location was on the tongue (n = 41, 52.56%), followed by the palate and lip (n = 13, 16.67%), gingiva (n = 6, 7.69%), jugal mucosa (n = 3, 3.85%) and finally the commissure (n = 2, 2.56%).

Regarding probable etiologies, authors seem to agree that the lesions are due to rashes directly associated with COVID-19 and the compromised state of the patients due to immunosuppression and stress. It has also been related to thrombotic alterations produced by the virus itself, without ruling out the eruptions associated with drugs for the treatment of COVID-(19).

The preferred treatment for the lesions is a localized one by using rinses. The rinses were based on antifungals (nystatin and fluconazole) (7,16,24), antibiotics (tetracycline, neomycin (11,13,26), ampicillin, sulbactam and clarithromycin (21), metronidazole (19)), antivirals (valacyclovir, acyclovir) (6,27), anesthetics (lidocaine) (11,16), topical corticosteroids (dextamethasone, triamcinolone, mometasone furoate) (11,15,25), chlorhexidine (7,6,15,19,10,23,24,25,27), 1% hydrogen peroxide, (7) and hyaluronic acid (6), also authors performed daily photobiomodulation (PBTM) theraphy (27). In some cases, they were treated with systemic medications such as corticosteroids (6,17,21), antifungals (acyclovir) (16) or analgesics (paracteamol) (20).

## Discussion

This study is carried out to review the new information that is emerging on oral lesions related to the COVID-19 disease. Being an emerging disease and becoming a global pandemic, there are constant changes and new information published/discovered about it. Chaux-Bodard *et al.* (12) described the first lesion associated with SARS-COV-2. Given the worldwide spread of the disease, the paucity of reports on oral manifestations suggests that oral lesions are rare in these patients (23). It is important to highlight the difference with SARS-COV-1 in which no associated oral lesions appear in the literature.

Regarding the etiology of the lesions, various hypotheses have been proposed. It has been discussed whether the lesions may be typical of COVID-19 or that COVID-19 is the promoter of the development of these oral manifestations. It now seems clear that coronavirus damage to respiratory organs and other organs could be related to the distribution of angiotensin converting enzyme 2 (ACE2) receptors. Therefore, cells that present the ACE2 receptor can become host cells for the virus and cause inflammatory reactions in oral organs and tissues (28,29,30). The recently published tropism of SARS-COV-2 to the tongue and salivary gland epithelium (31) suggest that the oral mucous membrane may be targeted by the virus. This leads to speculate that the development of oral manifestations may be directly associated with the COVID-19 infection (27).

In contrast, authors state that there is insufficient evidence to support an oral damage caused by SARS-COV-2 (23). Acute COVID-19 infection, together with associated therapeutic measures, could contribute to triggering alterations in the oral mucosa, which could probably cause various opportunistic fungal infections, recurrent oral herpes simplex virus infection, nonspecific oral ulcerations, dysgeusia, drug-associated eruptions, xerostomia related to decreased salivary flow, ulcers, and gingivitis. Also, most patients presented oral mucosal injury during the hospitalization period, supporting the hypothesis of coinfections, immunity impairment, or adverse reactions from medications to COVID-19 treatments (28).

On the other hand, it has been suggested that, among the probable causes of oral lesions, that they could be secondary to the deterioration of the immune system or due to disease treatments (28,17,21,32). The drug eruption can develop during the latency period. Drug hypersensitivity and urticaria have been reported by several patients and may not only be accidental, but may be related to the COVID-19-induced cytokine storm (21).

The immune status of the patient also influences the appearance of lesions. The signs and symptoms that some patients present (compatible with infections) can lead the patient to an immunosuppressed state that can trigger a reactivation of the Herpes simplex virus (32) or Varicella-Zoster virus (33).

Moreover, a concomitant bacterial superinfection may also occur (19,30) or the lesions may arise from an inflammatory reaction that induces vascular inflammation (30). In addition, injuries have been proposed to be a manifestation of the stress associated with the pandemic (11,29,32,33). The complications of the COVID-19 disease, the possibility of losing a friend or family member, the damage to economic conditions (32), work pressure and confinement increase stressful situations among patients (29). Therefore, the mental state of the patient should also be considered (32).

Regarding oral lesions, gingival alterations have occurred, such as desquamative (6) or necrotizing (19) gingivitis. Authors like Brandan *et al.* (34) hypothesized that periodontal pocket could be a favorable anatomical niche for the virus and thus acting as a reservoir for SARS-CoV-2. They justified this hypothesis, since periodontal pockets are ideal environments for subgingival bacterial biofilms, that interact with the supragingival oral cavity, mucosal tissues of the pocket and a peripheral circulatory system. Periodontal pockets have been found to harbor viral species such as the Herpes simplex viruses’ family; so, the same could happen with SARS-COV-2 (34).

Concerning the beginning of the oral manifestations, it varied considerably between the cases due to the lack of reference points. Days of laboratory testing, drug administration, hospital admission, hospital discharge, and respiratory and systemic manifestations were used as reference time points to describe the onset of oral symptoms. Although no case presented oral lesions prior to respiratory symptoms; although biases in data collection can be attributed (30).

The truth is that the SARS-COV-2 virus can cause oral lesions, which its incidence has probably been underestimated given the situation (27). Based on this, patients diagnosed with COVID-19 should undergo an oral inspection (16,22) in order to understand the pathobiology of these alterations (22). It is important that the new signs and symptoms are known, so that as we clinicians can report cases of COVID-19 (33). Dentists should be part of the multidisciplinary team for the diagnosis and treatment of these patients; as well as, on some occasions, being the first to identify the disease (28,29); as well as given the need for support, pain control, and quality of life (28).

This review has several limitations. First, the articles that relate oral lesions associated with COVID-19 disease are limited and also of low methodological quality. On the other hand, at the beginning of the pandemic, diagnostic tests were limited, so that some of the cases that are exposed are only suspected of infection by SARS-COV-2, based on the symptoms that the patients presented (6,18,25). Therefore, some lesions and symptoms could correspond to other frequent viral diseases (6,32). Another limitation is the difficulty for oral examination of patients, even some patients were visited indirectly (through photos or videoconferences) due to the high risk of contagion and the lack of protective equipment at the beginning of the pandemic. Furthermore, clinical imaging was not performed adequately due to the risk of contamination of photographic equipment (5,28). It is also worth noting that due to the severity of the disease, such an assessment becomes difficult for clinicians and, often without painful symptoms, patients end up reporting no injuries to the mouth (6,33).

## Conclusions

In conclusion, after conducting the literature review, it can be expected that the COVID-19 disease causes skin and mucosal lesions, probably as secondary manifestations. Although, more research of these clinical manifestations is needed to help us understand the disease.
